# Advances in *Leishmania* Vaccines: Current Development and Future Prospects

**DOI:** 10.3390/pathogens13090812

**Published:** 2024-09-20

**Authors:** Andreina Ayala, Alejandro Llanes, Ricardo Lleonart, Carlos M. Restrepo

**Affiliations:** 1 Centro de Biología Celular y Molecular de Enfermedades, Instituto de Investigaciones Científicas y Servicios de Alta Tecnología (INDICASAT-AIP), Panama City 0843-01103, Panama; aayala@indicasat.org.pa (A.A.); allanes@indicasat.org.pa (A.L.); rlleonart@indicasat.org.pa (R.L.); 2 Sistema Nacional de Investigación (SNI), Panama City 0801, Panama

**Keywords:** *Leishmania*, leishmaniasis, vaccine development, vaccine, immunity, treatment

## Abstract

Leishmaniasis is a neglected tropical disease caused by parasites of the genus *Leishmania*. As approved human vaccines are not available, treatment and prevention rely heavily on toxic chemotherapeutic agents, which face increasing resistance problems. The development of effective vaccines against human leishmaniasis is of utmost importance for the control of the disease. Strategies that have been considered for this purpose range from whole-killed and attenuated parasites to recombinant proteins and DNA vaccines. The ideal vaccine must be safe and effective, ensuring lasting immunity through a robust IL-12-driven Th1 adaptive immune response. Despite some success and years of effort, human vaccine trials have encountered difficulties in conferring durable protection against *Leishmania*, a problem that may be attributed to the parasite’s antigenic diversity and the intricate nature of the host’s immune response. The aim of this review is to provide a thorough overview of recent advances in *Leishmania* vaccine development, ranging from initial trials to recent achievements, such as the ChAd63-KH DNA vaccine, which underscores the potential for effective control of leishmaniasis through continued research in this field.

## 1. Introduction

Leishmaniasis is a disease caused by protozoan parasites of the genus *Leishmania*, transmitted by the bite of female phlebotomine sandflies [1]. Based on the complex interactions between the response of the host’s immune system and the implicated species of *Leishmania*, the main clinical manifestations or forms of the disease can be categorized as cutaneous (CL), mucocutaneous (MCL), and visceral leishmaniasis (VL) [2]. Ulcerative lesions that primarily affect the face, arms, and legs are the hallmark of CL, a form of the disease caused by species of two main *Leishmania* subgenera, *L.* (*Leishmania*) and *L.* (*Viannia*). These species are primarily found in Latin America, the Mediterranean Basin, the Middle East, and Central Asia. Although it has been reported that most cases of CL heal on their own [3], many may progress to MCL, even years after the primary lesions have resolved [4]. MCL is caused by the spread of parasites from the skin to the naso-oropharyngeal mucosa, where ulcerated lesions in the mouth can advance to the oropharynx and larynx, causing breathing problems, vocal cord damage, and even facial deformity [5]. MCL cases are caused by species of the subgenus *L*. (*Viannia)* (particularly *L. braziliensis*, *L. panamensis*, and occasionally *L. guyanensis*), which are exclusively present in Central and South America [6]. VL, by contrast, is predominant in Brazil, East Africa, and India [7], and it is caused by species of the subgenus *L*. (*Leishmania*) (mainly *L. infantum* and *L. donovani*) [8]. VL is the most severe form of the disease, and it can be fatal if left untreated. This form is characterized by persistent fever, hepatosplenomegaly, weakness, hypergammaglobulinemia, and pancytopenia [9]. Currently, over 1 billion people live in regions where leishmaniasis is prevalent and are at risk of infection. Furthermore, an estimated 700,000 new cases of VL and more than 1 million new cases of CL are reported each year [10].

Although leishmaniasis has devastating effects and is the second most common parasitic disease after malaria, it has long been overlooked and neglected worldwide [11]. Prevention and control of leishmaniasis poses significant challenges, including the lack of effective human vaccines and a limited set of effective antileishmanial drugs. In the absence of prophylaxis for humans, chemotherapy remains the main disease control strategy. Currently, treatment of the disease is mainly based on pentavalent antimonials, amphotericin B and its liposomal formulations, miltefosine, paromomycin, and pentamidine [12]. However, these drugs have several limitations, including high toxicity, many adverse effects, and prolonged parenteral administration, leading patients to discontinue treatment, which may eventually result in the selection of resistant strains [13].

Although licensed vaccines that are both safe and effective are still not available for human leishmaniasis, vaccination is still considered the most effective measure to control the disease [14]. The development of an antileishmanial vaccine is a highly promising goal and remains a global public health priority [15]. While extensive research has resulted in improved laboratory techniques to produce numerous preclinical vaccine candidates, the successful transition of *Leishmania* vaccines into human trials is still pending [16]. Over the years, only a few antileishmanial vaccines have reached the stage of clinical trials (Table 1), and only one therapeutic clinical trial is currently ongoing, having completed phase 2b on 28 February 2023 (Clinicaltrials.gov NCT03969134) [17]. In addition to the challenges faced in developing vaccines for human leishmaniasis, it is important to mention the control of canine leishmaniasis as part of broader eradication programs. Dogs are recognized as the primary reservoir hosts for *Leishmania* species that cause VL in humans, particularly in regions such as the Mediterranean, Latin America, and parts of Asia [18]. Moreover, the canine disease shares similarity with human VL and provides a model to study the immunopathogenesis of *Leishmania* infection [19]. As a result, vaccination strategies targeting dogs have been implemented in several countries as a crucial measure to reduce the spread of the disease [20]. The success of these canine vaccination programs has not only helped to reduce the incidence of leishmaniasis in dogs but has also contributed to lowering the transmission risk to humans. Additionally, insights gained from canine vaccine trials, including immune response, safety, and efficacy, can inform and accelerate the advancement of human vaccines, enhancing the overall effectiveness of vaccination programs aimed at eradicating leishmaniasis. Therefore, the present article aims to provide a comprehensive overview of the recent advances in the field of *Leishmania* vaccine development, providing an in-depth examination of the current status of each vaccine candidate that has entered clinical trials.

## 2. Immunological Landscape of *Leishmania* Infection

### 2.1. Host Immune Response against Leishmania

Resistance and susceptibility to *Leishmania* infection are influenced by several factors, including the species of the parasite, the location and number of vector bites, the inoculum size, and the host’s immunological status and response [35]. During infection, the parasites interact with multiple cells of the innate immune system, modulating their functions and phenotypes, as well as the successive adaptive response. The phagocytic cells at the inoculation site are mainly neutrophils and macrophages [36]. The parasites are phagocytosed by neutrophils, which in turn are phagocytosed by macrophages. Within macrophages, phagocytosed parasites establish themselves in compartments known as parasitophorous vacuoles. The surface of *Leishmania* parasites is covered by a layer of lipophosphoglycan (LPG) molecules that inhibit the fusion between the parasitophorous vacuole and lysosomes within the macrophage, preventing the formation of phagolysosomes and complement complexes [37]. This allows parasites to resist degradation within the vacuole and proliferate within the host’s macrophages, establishing infection. 

**Figure 1:**
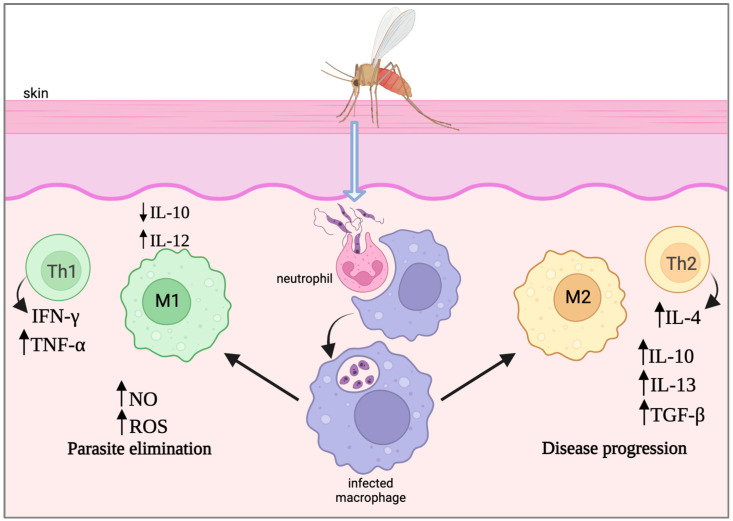
Th1/Th2 immune response to *Leishmania* infection. Infection of macrophages with *Leishmania* can promote the development of two distinct macrophage activation profiles known as M1 (green) and M2 (yellow), associated with either a Th1 or Th2 immune response, respectively. These scenarios are characterized by a different set of cytokines and ultimately lead to parasite elimination (Th1) or parasite progression (Th2). IL, interleukin; IFN-γ, interferon gamma; TNF-α, tumor necrosis factor alpha; NO, nitric oxide; ROS, reactive oxygen species; TGF-β, transforming growth factor beta. Created with BioRender.com.

The T helper lymphocytes play a critical role in coordinating the immune response against *Leishmania* in mammalian hosts and may provide direct protective immunity [38]. The direction of their divergence towards a Th1 or Th2 response has been shown to be a major factor in disease progression. The effective control of infection by *Leishmania* relies primarily on the IL-12-driven Th1-type immune response. Activation of macrophages to eliminate parasites is achieved through the secretion of interferon-γ (IFN-γ) and tumor necrosis factor alpha (TNF-α) by CD4^+^ T cells and antigen-specific CD8^+^ T cells [39]. The release of these essential cytokines, along with others such as IL-23, leads to the production of nitric oxide (NO) and reactive oxygen species (ROS) by macrophages (Figure 1). This type of classical macrophage activation profile, known as M1, has been reported to ultimately lead to immunity against reinfection [40]. On the other hand, disease progression was found to be largely influenced by the production of IL-4-driven Th2-associated cytokines, namely IL-4, IL-10, IL-13, and transforming growth factor beta (TGF-β) [41]. This response is associated with an anti-inflammatory phenotype and may promote an alternative macrophage activation profile known as M2, favoring parasite persistence (Figure 1). Unlike Th1, the Th2 immune response does not tend to neutralize intracellular parasites, leading to their dissemination and resulting in disease progression. The Th2 immune response fails to provide protective immunity against reinfection [42].

Conflicting functions of CD8^+^ T cells have been observed, which appear to be associated with the clinical presentation of the disease and the causative *Leishmania* species. Several studies have confirmed the protective role of these cells by reporting high levels of IFN-γ production or the direct killing of parasitized macrophages [43]. Studies in murine models have reported that the role of CD8^+^ T cells during *L. major* infection depends on the parasite infective dose. IFN-γ-producing CD8^+^ T cells appear to have a greater impact on steering CD4^+^ T cells towards a Th1 response in low parasite doses [44]. Furthermore, CD8^+^ T cells also seem to confer protection against reinfection in mice [45,46].

While the previously described roles of Th1 and Th2 immune responses appear to be valid for some Old World *Leishmania* species, such as *L. major*, significant differences have been reported for other species and the infection they cause in humans [47]. For instance, it has been shown that species of the *L.* (*Viannia*) subgenus promote mixed Th1/Th2 hyperinflammatory responses in humans [48,49]. Similarly, several studies have highlighted the contradictory roles of CD8^+^ T cells in infections caused by *L.* (*Viannia*) species. Some studies have shown that exacerbated activity of CD8^+^ T cells with poorly regulated responses may be associated with disease progression and evolution to MCL [50], while finely regulated CD8^+^ T cell responses are important for disease control in *L. braziliensis* [51,52] and for immune protection against *L. panamensis* [53].

In general, cells of the adaptive immune system play a dual role during *Leishmania* infections, as they can either boost the immune system to eliminate the parasite and establish a lasting response against reinfection or favor disease progression and strengthen its pathogenesis [54]. However, in contrast to other parasitic infections, individuals who successfully recover from leishmaniasis can develop an effective immune response that protects against reinfection [55], and a robust immune response can significantly contribute to the resolution of the disease. This highlights the significant involvement of immunological mechanisms in influencing the course of the disease, thereby justifying the potential of vaccination as a preventive strategy.

### 2.2. Mechanisms of Immune Evasion by Leishmania Parasites

*Leishmania* employs various mechanisms to evade the host’s immune response, significantly impacting the course of infection. One strategy involves the modification of the complement system and escaping phagocytosis. *Leishmania* can resist complement-mediated lysis through various mechanisms, such as preventing the formation of the membrane attack complex and utilizing surface molecules like LPG [56]. Additionally, in the promastigote stage, the parasite has been shown to have high expression of protein kinases which help to deactivate the classical and alternative complement pathways by C3, C5, and C9 phosphorylation [57]. Importantly, the zinc-dependent metalloprotease glycoprotein 63 (gp63) or leishmanolysin, a surface-expressed glycoprotein, has been shown to be crucial for resisting complement lysis by preventing the formation of C5 convertase [58]. This allows the parasite to effectively enter and survive within macrophages, while avoiding opsonization and subsequent phagocytosis, facilitating a “silent entry” into these immune cells.

Another critical aspect of immune evasion by *Leishmania* is the alteration of Toll-Like Receptor (TLR) pathways. TLRs are receptors expressed by cells of the innate immune system and are vital for recognizing pathogen-associated molecular patterns [59]. *Leishmania* has been shown to manipulate multiple TLR pathways to suppress pro-inflammatory responses [60]. For instance, the interaction of *L. major*’s LPG with TLR2 triggers the release of cytokines like TNF-α and IL-12, which are essential for an effective immune response. However, the parasite counters this by recruiting suppressors that inhibit these pathways [61]. *Leishmania* has also evolved mechanisms to manipulate TLR4 signaling to favor its establishment. During *L. major* infection, serine protease inhibitors block neutrophil elastase-mediated TLR4 activation, thereby hindering the uptake and killing of the parasite by the host’s macrophages [62]. Additionally, *L. mexicana* takes advantage of TLR4 signaling to impair IL-12 production by macrophages, further promoting its survival [63]. These findings suggest that various *Leishmania* species utilize distinct strategies to manipulate various TLR pathways, facilitating the parasite’s establishment and persistence within the host.

Another well-documented strategy employed by *Leishmania* is the prevention of the fusion of phagosomes with lysosomes within macrophages, thus avoiding the hostile environment that is normally lethal to pathogens [64]. By inhibiting endosome maturation and phagosome acidification, *Leishmania* can maintain a niche conducive to its survival. These parasites can also undermine host immune responses through defective antigen presentation and co-stimulation, which diminishes T cell activation. *Leishmania* parasites interfere with the expression of MHC class II molecules and co-stimulatory molecules, such as B7-1 and CD40, on macrophages, thereby impairing T-cell-mediated immunity [65]. Furthermore, *Leishmania* alters host cell signaling by disrupting pathways critical for macrophage activation, including the JAK/STAT and MAPK pathways, which are necessary for producing reactive oxygen species and nitric oxide [66]. Lastly, *Leishmania* modulates cytokine and chemokine expression to skew the immune response towards a Th2 phenotype, which is more favorable for its persistence [67]. This manipulation includes inducing IL-10, which further suppresses pro-inflammatory cytokines essential for clearing the infection; thus, the parasite effectively creates an immunosuppressive environment that favors its survival and propagation.

*Leishmania’*s diverse immune evasion strategies underscore its sophisticated adaptations to survive within the host. By exploiting these pathways, the parasite creates a favorable environment for its persistence, making the development of effective vaccines and therapies that can overcome these immune defenses challenging. This is why achieving concomitant immunity is crucial for vaccine development. The concept of “concomitant immunity” refers to a state of ongoing subclinical chronic infection with persistent low levels of parasites that induces a robust immune response [68]. This suggests that successful immunity against *Leishmania* infections relies not only on the presence of central memory T cells but also on the induction of specific subsets of effector T cells and tissue-resident memory (T_RM_) cells. The parasite’s ability to subvert host immune mechanisms, such as impairing antigen presentation and altering cytokine profiles, complicates the production of T_RM_ cells [69]. Therefore, to create an effective vaccine, it is essential to stimulate memory T cells and also ensure the development of subsets of effector T cells, such as Ly6C^+^ T cells, which are vital for protection against the parasite transmitted via sandfly bites [70]. The lessons learned from natural infections, such as those induced by leishmanization, highlight the need for vaccines to mimic this protective environment by ensuring that they promote the development of T_RM_ and effector Ly6C^+^ T cells [68]. Without these specific immune responses, vaccines may fail to provide adequate protection in real-world settings where vector transmission occurs, thereby limiting their efficacy.

### 2.3. Immune Dynamics of Leishmania–HIV Coinfection

Coinfection of *Leishmania* with HIV poses a special challenge due to the overlapping interaction of pathogen-specific immune responses. Although coinfection studies have mostly focused in determining viral/parasitic loads and response to treatments rather than changes in immune response [71], it has been shown that *Leishmania* and HIV-1 concomitant infection enhances the replication and pathogenicity of both pathogens [72,73]. Clinical and experimental studies suggest that the chronic immune activation mediated by TNF-α and IL-1α during *Leishmania* infection can upregulate HIV-1 expression and replication in host cells, leading to an increased HIV load and accelerated progression to AIDS [72,74,75]. Moreover, HIV-1 modulates cytokine production in response to *Leishmania*, shifting the immune response towards a Th2 profile, which is less effective at controlling *Leishmania* infection [71]. This shift contributes to increased susceptibility to *Leishmania*, more severe disease, and frequent reactivations in coinfected patients [76]. Additionally, the HIV-1 Tat protein further exacerbates *Leishmania* proliferation by promoting the secretion of cytokines that support parasite growth, such as prostaglandin E2 and TGF-β1 [77]. These findings highlight the complex bidirectional relationship between these pathogens, with *Leishmania* not only promoting HIV replication but also experiencing enhanced intracellular growth due to HIV-mediated immune impairment.

When designing antileishmanial vaccines for HIV-positive patients, a careful consideration of the immune system modulation is crucial. In general, HIV-infected individuals often have a diminished immune response to vaccines due to reduced CD4^+^ T cell counts [78]. Additionally, the HIV-induced dysregulated cytokine production biased towards a Th2 response worsens the *Leishmania* coinfection scenario [71]. Consequently, a vaccine must be potent enough to elicit a protective response even in the context of immune suppression, promoting a strong Th1 immune response. However, antigen and adjuvant formulations should be carefully selected in order to avoid an excessive immune activation that could potentially increase HIV viral loads. Regarding the type of vaccine, live-attenuated vaccines may pose a risk to immunocompromised patients due to the potential for uncontrolled replication. For this reason, subunit or killed vaccines may be safer alternatives. Despite concerns about depressed immunity, existing vaccines like those for influenza and *Streptococcus pneumoniae* have demonstrated protective benefits even in advanced HIV patients, supporting the evaluation of therapeutic *Leishmania* vaccines in this population [79,80]. However, detailed studies will be necessary to determine the optimal timing and dosage, particularly for those with advanced HIV. Safety concerns, while important, should not impede the evaluation of therapeutic vaccines, especially in HIV patients who are virally suppressed and on stable antiretroviral therapy. Evidence with vaccine formulations for other pathogens suggests that inactivated vaccines have similar safety profiles in both HIV-infected and uninfected individuals, and live-virus vaccines may be safely administered in individuals with well-controlled HIV loads and adequate CD4^+^ T cell counts [79].

## 3. First-Generation Antileishmanial Vaccines

### 3.1. Whole-Killed Parasites

First-generation vaccines are developed using whole-killed or live-attenuated parasites as a means of inducing broad immunity [81]. The first-generation vaccines against leishmaniasis centered around whole-killed parasites. These vaccines were developed due to their simplicity and low production cost, making them suitable for widespread distribution in developing countries. These types of antileishmanial vaccines were the first to be highly successful in providing immunity in animal models [82]. Leishvaccine was one of the first vaccines of this subgroup, made with an antigenic preparation of whole-killed *L. amazonensis* (strain IFLA/BR/1967/PH8) with Bacillus Calmette–Guérin (BCG) as an adjuvant, developed for the prevention of visceral canine leishmaniasis [21]. Leishvaccine was documented to induce initial alterations in the innate immune system mediated by neutrophils and eosinophils. These were followed by modifications in monocytes and activation of CD4^+^ T cells, CD8^+^ T cells, and B lymphocytes. It also prompted a mixed cytokine profile including IFN-γ and IL-4 [22].

Another vaccine made with autoclaved-killed *L. major* with BCG was assessed in phase I and II clinical trials among non-endemic healthy participants, showing safety but limited leishmanin skin test (LST) conversion and IFN-γ production [23]. Further investigations in CL endemic areas indicated minimal LST conversion, while a booster dose of autoclaved-killed *L. major* with BCG in Sudan resulted in a significant reduction in VL incidence among LST-converted individuals [83].

A different vaccine combining *L. mexicana* and *L. amazonensis* with BCG showed promising results by notably decreasing the occurrence of CL in Ecuadorian children in a year-long double-blind controlled field study [24]. To assess the duration of this protection, the study was modified to continue for an additional 48 months. The authors found the incidence of CL stayed lower in the vaccinated group, compared to the control group, up until 18 months. However, during a follow-up of 24–60 months, no significant differences were found between groups. Therefore, it was concluded that booster doses would be necessary to maintain immunity. Shortly after, in a multicenter randomized clinical trial involving 11,532 Venezuelan patients with localized CL, a vaccine with the same formulation demonstrated efficacy with minimal side effects and potentially low production costs [25]. In another study, the authors also conducted immunotherapy using pasteurized-killed *L. braziliensis* promastigotes and viable BCG, which proved successful in treating severe forms of CL, previously documented to be unresponsive to conventional chemotherapy in Venezuela [26]. Patients suffered minimal side effects and were free of active lesions for at least 10 months; however, no follow-up studies on this vaccine have been published. Overall, whole-killed promastigote vaccines seem to be a safe and cost-effective approach; nevertheless, further exploration of different adjuvants may potentially enhance their efficacy. However, the quality assurance and other procedures that may be required for the formal approval of such type of vaccines by regulatory agencies might be complex and cumbersome, limiting their commercial implementation [84].

### 3.2. Live-Attenuated Parasites

Based on the employed attenuation procedure, live-attenuated parasites can be divided into two categories: genetically defined or undefined parasites. The latter can be generated through various methods such as laser irradiation, chemical mutagenesis, or long-term in vitro cultivation [85]. However, undefined attenuation can result in a diminished ability to induce protective immunity either due to the failure of such strains to establish a subclinical infection or the loss of critical antigen epitope expression [86]. Alternatively, a defined genetic modification of the *Leishmania* genome can be achieved by specific mutagenesis, where a targeted gene is disrupted through processes such as homologous recombination or the novel CRISPR/Cas technology [87]. This strategy allows the selection of parasites that have lost the ability to encode one or more essential genes usually associated with virulence or long-term survival.

The most extensively studied live-attenuated vaccine candidate against *Leishmania* is indeed an example of a defined genetically modified parasite in which the *centrin* 1 (*Cen*1) gene has been inactivated using different approaches. *Cen*1 is a calcium-binding protein associated with the basal body in *Leishmania*, which plays a role in cell division through centrosome duplication and segregation [88]. Even though the inactivation of *Cen*1 does not seem to impact promastigotes in vitro, it interferes with the growth of amastigotes [89,90,91]. Indeed, axenic *L. donovani* amastigotes with a defective *Cen*1 gene exhibited cell cycle arrest at the G2/M phase, suggesting a failure in basal body duplication and cytokinesis [92]. Ultimately, this results in the formation of multinucleated cells, a condition that may activate the programmed cell death pathway of the parasite. One of the disadvantages of these initial attempts to generate *Leishmania Cen*1 knockouts was the inclusion of antibiotic resistance genes, associated with the plasmids and molecular biology constructs regularly used in the attenuation protocols [92]. The inclusion of antibiotic resistance genes in any attenuated vaccine raises concerns and is not deemed acceptable for human testing according to regulatory agencies [93]. To address this issue, recent attempts have focused on the use of the CRISPR/Cas technology for generating *Cen*1 knockouts. Advances in the CRISPR/Cas technology have revolutionized genome-wide loss-of-function screening in *Leishmania* parasites, eliminating the need for DNA double-strand breaks, homologous recombination, or donor DNA. A recent study successfully obtained an attenuated *Cen*1 knockout strain of *L. major* (Lm*Cen*^−^/^−^) without the presence of selection markers or off-target effects. This knockout strain was able to provide protective immunity in murine models after being challenged with *L. major*-infected sandflies [70]. These results highlight Lm*Cen*^−/−^ as a promising candidate for future human trials. This approach, while promising, may need more research and experimental validation for different species, since a recent report using *L. braziliensis* found that knocked-out parasites for the same gene were not able to induce protection in vaccinated BALB/c mice. However, using the same animal model, these authors showed that centrin-deficient *L. donovani* parasites effectively induced protection against *L. braziliensis* challenge [94].

Although live-attenuated parasites have the advantage of containing all possible antigens and have been shown to yield significant protection in murine models, one of their disadvantages is the potential for reversion, which may cause the return to a virulent state. Indeed, arbitrary mutations have often been observed in live-attenuated parasites [95,96,97], especially when using physical or chemical methods of attenuation. An alternative to artificial attenuation of parasites can be naturally nonpathogenic species of *Leishmania*, such as *L. tarentolae*. These species closely resemble pathogenic strains but lack virulence genes that cause disease, eliminating the need for attenuation [98]. Studies have shown that immunizing with *L. tarentolae* may trigger protective immune responses against *L. donovani* in mice [99]. More specifically, *L. tarentolae* seems to stimulate the maturation of dendritic cells, prompting T cell proliferation and increasing IFN-γ production, thus directing CD4^+^ T cells towards a protective response [99]. Another vaccine candidate based on genetically modified *L. tarentolae* was shown to protect BALB/c mice against *L. infantum* infection [100]. This vaccine expressed the *L. donovani* A2 antigen along with cysteine proteinases (CPA and CPB without its unusual C-terminal extension) as a tri-fusion gene. The authors showed that the generation of immunity in treated BALB/c mice was associated with the induction of a Th1-type immune response with high levels of IFN-γ production before and after challenge. In a very recent publication, transgenic *L. tarentolae* strains engineered to express gamma glutamyl cysteine synthetase (γGCS) derived from *L. donovani*, *L. major* or *L. mexicana* were developed for assessing their protective efficacy against both CL and VL [101]. The vaccine, administered in two doses, notably reduced parasite levels for *L. major* and *L. donovani* compared to their respective control groups. The protection observed was linked to a Th1 immune response in *L. major* challenge and a mixed Th1/Th2 response in *L. donovani* infection.

## 4. Second-Generation Antileishmanial Vaccines

Second-generation antileishmanial vaccines refer to recombinant *Leishmania* antigens that are single peptides or polypeptides produced using genetically engineered viruses and bacteria [102]. These antigens are characterized by their high level of purification, which enables standardization and large-scale production. Furthermore, they are cost-effective and have excellent reproducibility. Many of the second-generation vaccines against *Leishmania* have been designed specifically for dogs. Dogs play a crucial role in the transmission of *Leishmania* infections, serving as the primary reservoir hosts for various species and can harbor the infection even when asymptomatic [103]. This facilitates the spread of the disease in endemic areas, through sandfly vectors that bite infected dogs and subsequently transmit the parasite to humans and other animals [104]. The close association dogs have with humans underscores the importance of controlling the disease in canine populations to reduce human infection risks. The development of vaccines for dogs, alongside those for humans, reflects a comprehensive approach to managing and controlling leishmaniasis; by targeting the canine reservoirs, public health initiatives can break the transmission cycle, thereby protecting both animal and human populations from this disease [105]. Leishmune (Zoetis Industria de Produtos Veterinarios LTDA, São Paulo, Brazil) and CaniLeish (Virbac, Carros, France) are examples of second-generation veterinary licensed vaccines that have been commercialized in Brazil and Europe, respectively. These vaccines have proven to be effective in providing protection to canines against *Leishmania* and interrupting the transmission of the parasite from dogs to humans [20]. Leishmune’s formulation is based on the fucose–mannose ligand (FML) of *L. donovani*, a major antigenic complex whose main antigen is NH36 (an enzyme that is essential for DNA synthesis in the parasite) along with saponin as an adjuvant [27]. In two phase III field trials conducted in endemic regions of Brazil, Leishmune demonstrated efficacy, with 92–95% of vaccinated dogs being protected against canine VL. The immunogenicity was demonstrated by 98% of FML seroconversion, an increase in absorbencies, 82.7% DTH positive reactions, and an increase in skin test size diameters [106]. An average increase in CD8^+^ total lymphocytes in blood, the sustained proportions of CD4^+^ T cells, and the average increased proportions of CD21^+^ B lymphocytes were also reported [106]. However, due to the lack of sample randomization and blinded evaluation of trial individuals, the results were not fully validated. Consequently, in 2014, the Brazilian Ministry of Agriculture withdrew the production and marketing license of Leishmune, citing insufficient evidence of effectiveness from phase III trials.

The CaniLeish vaccine, released in 2011, is composed of purified excreted–secreted proteins of *L. infantum* (LiESP), adjuvanted with saponin. In field studies, vaccinated dogs developed a Th1 immune response within three weeks, and the vaccine exhibited a remarkable infection protection rate of 99.4% [28]. A study conducted in 2020 found the CaniLeish vaccine to be safe in dogs from an endemic area [29]. Nevertheless, during the first year after vaccination, no discernible difference in the number or severity of active *L. infantum* infection cases was observed between the vaccinated and control groups. Leish-Tec is another veterinary vaccine comprising recombinant protein A2 sourced from *L. donovani* amastigotes, along with saponin as an adjuvant. A study evaluating Leish-Tec highlighted its efficacy not only as a prophylactic vaccine but also in immunotherapeutic applications [107]. Additionally, vaccination substantially diminished the susceptibility to infection of dogs by sandflies [30]. Another veterinary vaccine called LetiFend incorporates Protein Q, a chimeric protein consisting of five antigenic fragments from four different *L. infantum* proteins (histone H2A and ribosomal proteins LiP2a, LiP2b, and LiP0). Its efficacy was thoroughly examined in a large sample of dogs, encompassing various breeds and age groups, over a 2-year field study [18]. The results clearly demonstrated that vaccination significantly reduced clinical signs associated with the progression of the disease.

Regarding antileishmanial vaccines for humans, recombinant antigen vaccines LEISH-F1 (formerly Leish-111f), LEISH-F2, and LEISH-F3 have been developed and studied by the Infectious Disease Research Institute (IDRI) in Seattle, USA. LEISH-F1, a recombinant polyprotein consisting of *L. major* homologue of eukaryotic thiol-specific antioxidant (TSA), *L. major* stress-inducible protein 1 (LmSTI1), and *L. braziliensis* elongation and initiation factor (LeIF), was evaluated following emulsification with monophosphoryl lipid A (MPL-SE) as an adjuvant [31]. LEISH-F1 + MPL-SE showed promise in the treatment of CL and ML patients while inducing protective immunity in healthy volunteers. LEISH-F2, an improved version of LEISH-F1, excludes the N-terminal histidine tag of the recombinant polyprotein of LEISH-F1 and substitutes Lys274 of this polyprotein by glutamine, showing potential therapeutic effects on CL patients in a phase II clinical trial when combined with MPL-SE adjuvant [32]. LEISH-F3, another multicomponent vaccine, combines nucleoside hydrolase (NH) from *L. donovani* and sterol 24-c-methyltransferase (SMT) from *L. infantum* with the TLR-4 ligand glucopyranosyl lipid A in a stable oil-in-water nano-emulsion (GLA-SE) as an adjuvant. In clinical trials, LEISH-F3 demonstrated a robust immune response induction against VL in healthy adults [33]. Several other subunit or recombinant candidates have been extensively studied for second-generation vaccine development. For example, gp63has been investigated in various immunization schemes [108,109]. However, findings from animal models did not consistently translate into protective immune responses in humans. Multiple studies have reported the inability of gp63 to activate peripheral blood mononuclear cells (PBMCs) from patients treated for leishmaniasis [110,111,112]. Other much-investigated antigens include *Leishmania* elongation and initiation factor [113], kinetoplastid membrane protein 11 [114], amastigote-specific protein A2 [115], cysteine proteinase B [116], and K26/HASPB [117]. Recently, Poly-T Leish, a polyepitope T cell antigen consisting of epitopes of *L. infantum* proteins CPA, CPB, PSA-50S, and A2, was tested as a candidate against VL in BALB/c mice [118]. Immunization with this polyepitope antigen triggered a Th1 immune response which led to the activation of multifunctional T cells producing IFN-γ, TNF-α, and IL-2, as well as a decrease in IL-4 and IL-10. While most of these recombinant antigens have undergone evaluation in animal models to assess their immunogenicity and protective capabilities, only a limited number have advanced to clinical trials involving non-human primates, dogs, or early-stage human studies [119].

## 5. Third-Generation Antileishmanial Vaccines

With the purpose of improving the precision and effectiveness of vaccination, third-generation vaccines are under development. These vaccines include nucleic-acid-based formulations, based either in DNA or RNA, usually in prime–boost vaccination strategies [120]. DNA vaccines against *Leishmania* were originally designed with genes encoding individual antigens. The first DNA antileishmanial vaccine contained the gene encoding gp63. This vaccine demonstrated significant efficacy in protecting against *L. major* infection [121] and conferred partial protection against *L. mexicana* in murine models [122]. However, the *Leishmania* homologue for the receptors of activated C kinase (LACK) has been the most common antigen used for DNA vaccine development against both cutaneous (*L. major*) and visceral (*L. donovani* and *L. infantum*) leishmaniasis [123,124]. Nevertheless, these vaccines have displayed variable outcomes possibly due to differences in formulations.

To increase the ability of DNA vaccines of inducing immune responses, prime–boost strategies have been employed. Two main paradigms have been used in this case, namely, the same antigen can be used for both the initial and subsequent booster doses, or the immune system can be first primed with one antigen and then boosted with a different one [125]. A heterologous prime–boost approach utilizing the gp63 antigen and CpG- oligodeoxynucleotides (CpG-ODN) was tested on BALB/c mice [126]. This vaccination protocol induced robust cellular and humoral responses in mice after being challenged with *L. donovani* promastigotes. Additionally, BALB/c mice were also conferred protection from DNA encoding the A2 protein when challenged with *L. amazonensis* [127]. Furthermore, protection against *L. major* was effectively elicited also in BALB/c mice, along with a Th1 immune response by another TSA-based DNA vaccine [128].

As an alternative, several genes that encode different antigens have been combined to create DNA vaccines. One example is the HisAK70 DNA vaccine, encoding seven *Leishmania* antigens (H2A, H2B, H3, H4, A2, KMP11, and HSP70), which was administered subcutaneously to BALB/c mice [129]. HisAK70-immunized mice exhibited a strong Th1 immune response characterized by higher levels of IFN-γ, IL-12, and granulocyte macrophage colony-stimulating factor, as well as lower levels of Th2 cytokines (IL-4 and IL-10), which was associated to a resistant phenotype against *L. amazonensis* infection [129]. Another example is the ChAd63-KH vaccine [34], which uses simian adenovirus (ChAd63) and contains a novel synthetic gene (KH) encoding two *L. donovani* proteins, kinetoplastid membrane protein 11 (KMP-11) and hydrophilic acylated surface protein B (HASB). It has recently been shown that ChAd63-KH was able to efficiently elicit a broad variety of CD8^+^ T cells that are specific to *Leishmania* antigens in post-kala-azar dermal leishmaniasis (PKDL) patients in Sudan [34]. PKDL is a chronic skin condition that frequently appears after apparent cure from VL. It has also been reported that intramuscular doses of the vaccine were safe and effectively stimulated the production of IFN-γ and the activation of dendritic cells in patients [130]. This vaccine has shown promising immunogenic and safety results in a phase 1 clinical trial in the United Kingdom and in a phase 2a trial in Sudan [17]. Overall, DNA-based vaccines are stable, generate antigens over long periods of time, do not require adjuvants, can inexpensively be produced in large quantities, and seem to be effective [131]. However, further investigation is required as they raise some concerns around safety, such as the possibility of integration of DNA into the genomes of mammals which has the potential to cause cancer or trigger autoimmune diseases [132].

To the best of our knowledge, non-viral RNA-based antileishmanial vaccines have not been incorporated in clinical trials. However, given the success of mRNA vaccines against COVID-19, many researchers are now focusing on leveraging this promising platform to develop vaccines against parasitic infections. Like DNA-based vaccines, mRNA vaccines offer rapid, low-cost production and seem to elicit strong, durable immune responses, making them an attractive option for combating these complex diseases in the near future [133]. Although RNA-based vaccines for parasitic infections are still in early stages, a relatively recent study reported protection against *L. donovani* infection in mice vaccinated with an alphavirus-based RNA vaccine expressing the previously validated recombinant antigen LEISH-F2, followed by immunization with a defined subunit vaccine containing LEISH-F2 along with GLA-SE [134]. These results further illustrate the usefulness of RNA-based technology and heterologous prime–boost immunization approaches to induce defense against *Leishmania*.

## 6. Conclusions: Limitations and Future Prospects

In general, effective vaccine design and production depends on a multitude of factors including understanding the immunobiology of pathogen/host interactions, choosing appropriate vaccine candidates, and identifying the best adjuvant or delivery system. It also requires the ability to prime and maintain T cell responses specific to parasites, generate T lymphocyte cells with the proper effector functions, and induce effective antigen-specific antibody responses [135]. Furthermore, to efficiently evaluate vaccine efficacy and facilitate the transition from preclinical studies to human trials, it is essential to identify the key immune responses that indicate protection, as these are essential for achieving long-term immunity. In the case of *Leishmania* vaccines, the main goal is to elicit a strong Th1 memory response, promoting an early activation of IFN-γ-producing effector T cells at the challenge site. These responses are essential for mediating infection control and parasite killing, as well as disrupting the development of parasite persistence. The immunological protective mechanism of leishmaniasis is still not clear for all pathogenic species, despite advances in our understanding of immune regulatory pathways established after infection and the critical role that cell-mediated immunity plays in host protection. The failure of human vaccine trials to provide long-term immunity against this sandfly-borne infection can be attributed to the parasite’s antigen diversity, the intricacy of the host’s immune response, and even to potential immunomodulatory effects of sandfly saliva during the early stages of infection. Differences in the virulence factors of species within this genus and the distinct immune responses they elicit could explain the variety of clinical manifestations of *Leishmania* infections.

Additionally, promising vaccine candidates face several challenges, including low protective immunity in killed vaccines and problems with live-attenuated vaccines that raise the possibility of vaccine-induced leishmaniasis in immunocompromised individuals. Since recombinant and nucleic-acid-based vaccines are still in the early stages of development, worries about potential side effects persist. Additionally, the adjuvant selected during vaccine development is important and varies depending on antigen type, administration route, and expected immune response. Another barrier in making an antileishmanial vaccine for humans that cannot be overlooked is the lack of financial incentives, which makes it difficult to attract industry interest. Since leishmaniasis is mainly prevalent in developing and poor nations, it is challenging to recover the very high costs associated with research, development, and clinical trials of a vaccine candidate. As leishmaniasis is a neglected disease, the same situation is affecting the development of new therapeutic agents. Although there is currently no approved *Leishmania* vaccine for humans, tremendous efforts are being made worldwide to develop multiple vaccine strategies with promising results. Several vaccine candidates have been incorporated to clinical trials and continue to be evaluated presently at different clinical phases. In the meantime, animal antileishmanial vaccination is believed to be essential in halting the spread of the disease to humans in certain territories and species. Ultimately, we are optimistic leishmaniasis can eventually be controlled if more resources can be dedicated to the complicated and challenging process of vaccine development.

## Figures and Tables

**Table 1:** *Leishmania* vaccines evaluated in clinical trials: information consulted until 30 June 2024.

Vaccine	Classification	Vaccine Antigen	Adjuvant	Target	Phase Reached	Major Findings	References
Leishvaccine	First generation	Whole-killed promastigotes of *L. amazonensis*	BCG	Dogs	III	Induced modifications in monocytes, activation of CD4^+^ T cells, CD8^+^ T cells, and B lymphocytes. It also prompted a mixed cytokine profile including IFN-γ and IL-4.	[21,22]
Autoclaved *Leishmania*	First generation	Killed *Leishmania* spp.	BCG	Humans	III	Minimal LST conversion in participants and significant reduction in VL incidence among LST-converted individuals.	[23,24,25,26]
Leishmune	Second generation	FML	Saponin	Dogs	III	Between 92 and 95% of vaccinated dogs were protected against canine VL.	[27]
CaniLeish	Second generation	LiESP	Saponin	Dogs	III	Vaccinated dogs developed a Th1 immune response within three weeks, and the vaccine exhibited a protection against infection rate of 99.4%.	[28,29]
Leish-Tec	Second generation	*L. donovani* A2 protein	Saponin	Dogs	III	Vaccination of infected healthy animals significantly reduced clinical progression and decreased mortality.	[30]
LetiFend	Second generation	*L. infantum* proteins (H2A, LiP2a, LiP2b, and LiP0)	None	Dogs	III	Overall efficacy in the prevention of confirmed cases of canine leishmaniasis in endemic areas with high disease pressure was shown to be 72%.	[18]
Leish-F1	Second generation	TSA, LmSTI1, and LeIF	MPL-SE	Humans	I	The vaccine was safe and well tolerated by participants and induced T cell production of IFN-γ and other cytokines in response to stimulation with the antigen.	[31]
Leish-F2	Second generation	TSA, LmSTI1, and LeIF	MPL-SE	Humans	II	Showed potential therapeutic effects on CL patients when combined with the adjuvant.	[32]
Leish-F3	Second generation	NH36 and SMT	MPL-SE and GLA-SE	Humans	I	Subjects vaccinated with Leish-F3 and GLA-SE had significant levels of antigen-specific IgG antibodies in their serum, along with IFN-γ, TNF, and IL-2 secretion in response to the antigen.	[33]
ChAd63-KH	Third generation	KMP-11 and HASPB	None	Humans	II	It elicited a variety of CD8^+^ T cells specific to *Leishmania* antigens in PKDL patients. Vaccination was safe and effectively stimulated the production of IFN-γ and the activation of dendritic cells.	[34]

BCG, Bacillus Calmette–Guérin; FML, fucose–mannose ligand; LiESP, *L. infantum* excreted–secreted protein; TSA, thiol-specific antioxidant; LmSTI1, *L. major* stress-inducible protein 1; LeIF, *L. braziliensis* elongation and initiation factor; MPL-SE, monophosphoryl lipid A; NH, nucleoside hydrolase; SMT, sterol 24-c-methyltransferase; GLA-SE, glucopyranosyl lipid A stable oil-in-water nano-emulsion; KMP-11, kinetoplastid membrane protein 11; HASPB, hydrophilic acylated surface protein B.
